# Sarcoid-Like Reactions in Breast Cancer Patients: A Report of Two Cases

**DOI:** 10.7759/cureus.64096

**Published:** 2024-07-08

**Authors:** Nikolaos Marinos, Michail Visvikis, Vasiliki E Georgakopoulou, Fotios Drakopanagiotakis, Paschalis Steiropoulos

**Affiliations:** 1 Department of Respiratory Medicine, Medical School, Democritus University of Thrace, Alexandroupolis, GRC; 2 Department of Pathophysiology/Pulmonologist, Laiko General Hospital, Athens, GRC

**Keywords:** lymph nodes, granuloma, sarcoidosis, sarcoid-like reactions, breast cancer

## Abstract

Sarcoid-like reactions (SLR) in patients with malignancies are a relatively common finding. Defined by the presence of non-caseating granulomas, SLR does not meet the clinical criteria for classic sarcoidosis. In cancer patients, SLR often presents a challenging differential diagnosis, as it must be distinguished from disease progression due to malignancy. We present two cases of SLRs associated with breast cancer, underscoring the need for heightened vigilance among physicians. SLR should always be considered a potential diagnosis in these patients, with histological confirmation being essential for accurate identification.

## Introduction

Sarcoid-like reaction (SLR) is characterized by the formation of non-caseating granulomas in genetically susceptible patients, caused by various factors, but their clinical characteristics do not meet the diagnostic criteria for sarcoidosis [1]. The primary distinction between SLR and sarcoidosis is the absence of systemic symptoms in SLR [2]. While granulomas in sarcoidosis are marked by the presence of T-cells and antigen-presenting cells, SLR granulomas also exhibit B-cell involvement [3,4]. However, distinguishing between sarcoidosis and SLR based on histological images is challenging [5].

The development of SLR is attributed to malignancies, infections, and drugs, and it may be found in any lymph node, with intrathoracic lymph nodes being predominantly affected [1,2,6]. Malignancy-associated SLR is believed to result from tumor antigens stimulating T-cell-mediated immune responses, leading to the formation of non-caseating epithelioid cell granulomas [1]. SLRs may occur alongside solid tumors, in lymph nodes following the drainage pathway of the tumor and in distant locations [2,7].

Breast cancer is one of the most common malignancies associated with SLR, with some studies suggesting it may be the most prevalent solid tumor linked to SLRs. Other frequent malignancies include prostate cancer, lymphoma, non-small cell lung carcinoma (NSCLC), and skin cancer [1,3]. In cancer patients, chemotherapy and immunotherapy are additional potential causes of SLR [7]. Immunotherapy, particularly with immune checkpoint inhibitors, is often associated with SLR, though other drugs targeting different components of the immune system can also contribute [8].

This article presents two cases of SLRs occurring in breast cancer patients.

## Case presentation

Case 1

A 71-year-old female patient presented to our hospital with a dry cough that had begun three days prior to admission. She had been diagnosed with ductal carcinoma in situ (DCIS) of the right breast four months earlier. Immunohistochemical analysis was positive for progesterone receptor (PR) (80/100) and estrogen receptor (ER) (40/100) markers, and for erythroblastic oncogene B (cerbB2) (score 3+) oncoprotein. She had undergone a segmentectomy with lymphadenectomy, and histopathological examination of the axillary lymph nodes revealed metastatic disease in one of the four isolated lymph nodes. She was receiving systemic therapy with paclitaxel, pertuzumab, and trastuzumab.

**Figure 1:**
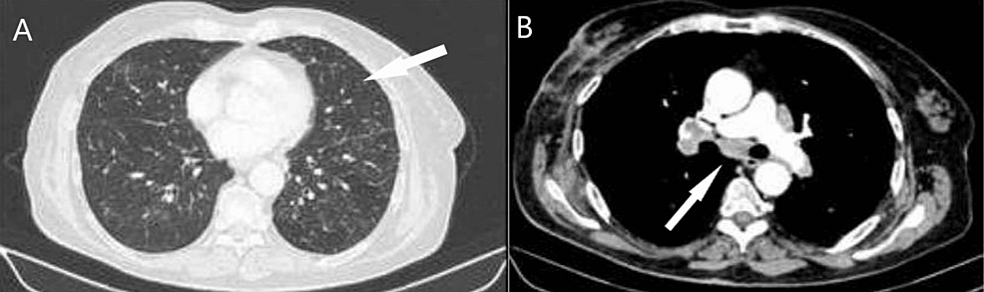
Chest CT. A: Chest CT revealed bilateral micronodules (arrow); B: Chest CT revealed enlarged mediastinal lymph nodes (arrow). CT, computed tomography

A lung computed tomography (CT) scan showed bilateral micronodules and enlarged mediastinal lymph nodes (Figure 1).

An abdominal CT scan revealed no pathological findings. To further evaluate the parenchymal pathology and the mediastinal and right axillary lymphadenopathy, a positron emission tomography-computed tomography (PET-CT) scan was performed. The PET-CT showed hypermetabolic mediastinal lymph nodes, mildly metabolic supraclavicular lymph nodes, and slight 18F-FDG uptake in the right axillary cavity adjacent to postoperative lesions and posterior to the pectoralis major. The lung micronodules also exhibited slight 18F-FDG uptake.

Pulmonary function tests indicated a restrictive pattern, with decreased forced expiratory volume in one second (FEV1), forced vital capacity (FVC), peak expiratory flow (PEF), and an FEV1/FVC ratio of 0.85. A Quantiferon-TB assay was positive. Bronchoscopy was performed, and microscopy and polymerase chain reaction (PCR) of the bronchoalveolar lavage (BAL) were negative for tuberculosis. The cytological examination of bronchial secretions revealed no malignancy. Pathological examination showed multinucleated giant cells and granulomas, findings indicative of an SLR.

Case 2

**Figure 2:**
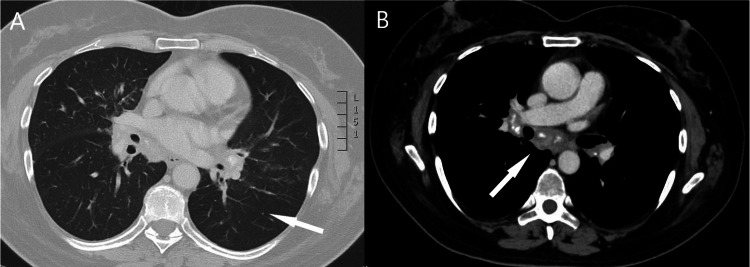
Chest CT. A: Chest CT revealed bilateral micronodules (arrow); B: Chest CT revealed enlarged mediastinal lymph nodes (arrow). CT, computed tomography

A 53-year-old female patient was admitted to the hospital for the investigation of lymphadenopathy and skin lesions. Histological examination of the skin lesions revealed granulomas. She was undergoing chemotherapy with docetaxel, trastuzumab, pertuzumab, and letrozole for invasive ductal carcinoma of the right breast. During her hospital stay, the tuberculin skin test (TST) and Wright test were negative, while her serum angiotensin-converting enzyme (sACE) level was elevated at 96 U/L. CT scans revealed small parenchymal nodules in the lungs and enlargement of mediastinal, hilar, and axillary lymph nodes (Figure 2).

A bronchoscopy revealed stenosis of the right medial bronchus, and a biopsy was performed. Cytological examination of the BAL fluid from the lingula, along with culture, microscopy, and PCR for Mycobacterium tuberculosis, as well as cultures for common pathogens, fungi, and mycobacteria, were all negative. Lung biopsy showed inflammatory infiltrations, numerous multinucleated giant cells, and Schaumann bodies, findings consistent with an SLR.

Pulmonary function tests indicated decreased FEV1, FVC, TLC, and an FEV1/FVC ratio of 0.73. Follow-up chest CT scans at three and eight months showed no significant changes in the lung nodules and lymph nodes.

## Discussion

Numerous studies have attempted to determine the temporal relationship between cancer diagnosis and the onset of SLR, with varying results. Most studies conclude that the majority of patients develop granulomatous lesions within the first five to six years following cancer diagnosis [2,3,5]. In our cases, SLR appeared to occur concurrently with or shortly after the cancer diagnosis, although we cannot precisely estimate the onset of lesion development. Besides cancer itself, malignancy treatments, including chemotherapeutics, are potential causes of SLR [2]. Τhe systemic therapy with paclitaxel, pertuzumab, and trastuzumab could potentially impact SLR by modulating the immune system. Paclitaxel, a chemotherapeutic agent, stabilizes microtubules and disrupts cell division, leading to apoptosis and enhancing immune responses, which may contribute to non-caseating granuloma formation seen in SLR​ [9]. Pertuzumab and trastuzumab, monoclonal antibodies targeting the HER2 receptor, inhibit HER2 signaling and promote antibody-dependent cellular cytotoxicity (ADCC), further activating immune cells such as T-cells and NK cells, which could increase the likelihood of developing SLR​​ [10]. It has been suggested that granulomatous reactions associated with malignancy correlate with a better prognosis and a lower risk of stage IV metastatic disease, particularly in patients with lymphoma and testicular cancer [3,11].

Most cases of malignancy-associated SLR present with mediastinal, hilar, and axillary lymphadenopathy. Additionally, pulmonary involvement, manifesting as pulmonary nodules and intra-abdominal lymphadenopathy, may occur in SLR patients [12]. However, remote sarcoid-like lesions, such as skin lesions or splenic granulomas, can complicate the diagnosis [2]. In our cases, patients exhibited characteristic nodular lesions in the lung parenchyma accompanied by pathological pulmonary function tests. Additionally, one patient showed signs of intra-abdominal lymphadenopathy and skin lesions.

SLRs have a heterogeneous presentation, affecting various systems and organs, thereby posing challenges for differential diagnosis. It is essential to distinguish granulomas caused by sarcoidal pathology from those induced by infections, drugs, or neoplasia. Understanding that SLR can present with diverse clinical manifestations is crucial. Therefore, thorough clinical, radiological, and histopathological examinations are necessary to exclude other causes of granulomatous diseases [4].

The presence of mediastinal lymphadenopathy discovered during staging or follow-up imaging in patients diagnosed with breast cancer presents a significant diagnostic challenge. In such cases, SLR should always be considered in the differential diagnosis, and histological confirmation is required to establish the correct diagnosis [1,2].

## Conclusions

The presented cases of SLR in breast cancer patients underscore the diagnostic challenges associated with this condition, particularly when mediastinal and pulmonary involvement is present. Despite extensive evaluations, pinpointing the exact onset of SLR lesions remains elusive. Both patients demonstrated characteristic lung nodules and lymphadenopathy, with one patient also presenting with skin lesions. Given the potential for SLR to closely mimic metastatic disease, it is imperative to include SLR in the differential diagnosis of lymphadenopathy in cancer patients. The differential diagnosis should encompass a broad spectrum of possibilities, including metastatic disease, other granulomatous diseases such as tuberculosis and sarcoidosis, and drug-induced granulomas. Histological confirmation through biopsy is crucial in distinguishing SLR from these other conditions. Histopathological examination can reveal non-caseating granulomas typical of SLR, which helps differentiate it from granulomatous infections that often present with caseating granulomas. Additionally, special stains and cultures may be necessary to rule out infectious causes. Drug histories should be meticulously reviewed to identify potential drug-induced etiologies. Comprehensive clinical, radiological, and histopathological evaluations are necessary to ensure an accurate diagnosis and appropriate management of SLR in oncology patients. Radiological imaging, including CT and PET scans, can aid in identifying characteristic patterns of SLR, though these findings are not definitive without histological correlation. Clinical correlation with patient history, presentation, and response to treatments is also vital. Accurate diagnosis is essential to prevent unnecessary treatments for presumed metastatic disease and to ensure that patients receive appropriate therapy for SLR.
